# Lifestyle impact and the biology of the human scrotum

**DOI:** 10.1186/1477-7827-5-15

**Published:** 2007-04-20

**Authors:** Richard Ivell

**Affiliations:** 1 Research Centre for Reproductive Health, and School of Molecular and Biomedical Science, University of Adelaide, SA 5005, Australia

## Abstract

The possession of a scrotum to contain the male gonads is a characteristic feature of almost all mammals, and appears to have evolved to allow the testes and epididymis to be exposed to a temperature a few degrees below that of core body temperature. Analysis of cryptorchid patients, and those with varicocele suggest that mild scrotal warming can be detrimental to sperm production, partly by effects on the stem cell population, and partly by effects on later stages of spermatogenesis and sperm maturation. Recent studies on the effects of clothing and lifestyle emphasize that these can also lead to chronically elevated scrotal temperatures. In particular, the wearing of nappies by infants is a cause for concern in this regard. Together all of the evidence indirectly supports the view that lifestyle factors in addition to other genetic and environmental influences could be contributing to the secular trend in declining male reproductive parameters. The challenge will be to provide relevant and targeted experimental results to support or refute the currently circumstantial evidence.

## Background

The exteriorization of the male gonads in a special sac called the scrotum is a uniquely mammalian feature, and one that at first glance requires some explanation. Surely, a trait that would place all of the genetic material essential for procreation in such an exposed situation, rather than protecting it deep within the organism (e.g. like the ovaries) must have some important selective advantage; otherwise why could it evolve and why has it not been selected against. The most plausible evolutionary explanation relates to the requirement of spermatogenesis for an optimum temperature lower than core abdominal temperature [1]. Certainly, the temporary exposure of the adult testes to mild warming (abdominal temperature) leads to marked disruption of spermatogenesis and/or male fertility [2,3]. Testicular descent into a scrotum evolved probably more than 150 million years ago concurrently with the acquisition by ancestral mammals of a regulated hyperthermia [1], which provided the abdominal organs with a controlled, stable body temperature of ca. 36–38°C. It is notable that in many mammals which have reverted to having abdominal testes, either there is a specialized blood supply acting as a heat exchanger to cool the abdominal gonad (e.g. whales), or the core temperature is itself relatively low 34–36°C (e.g. some insectivores) [1]. Although elephants appear to have abdominal testes as a primitive trait, there is recent evidence to suggest that they may have had an aquatic origin in the distant past [4], possibly at a time of early mammalian radiation, when the scrotal trait was not fully established in all mammalian lineages.

**Figure 1:**
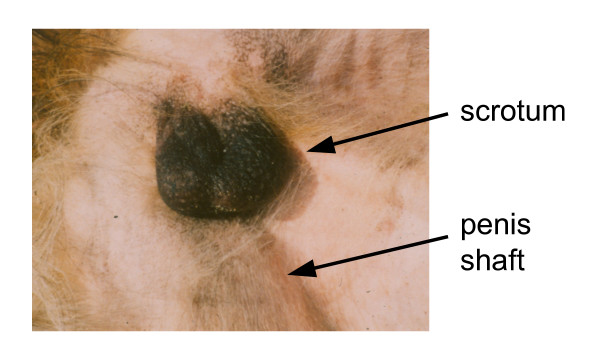
Scrotum of a dog to illustrate natural dark coloration and hairlessness to encourage heat loss. The dark colour also probably protects from UV irradiation.

Why a reduced scrotal temperature has selective advantage is not immediately clear, particularly since a number of animals (such as elephants, hyraxes and reptiles) appear to survive and reproduce with abdominal testes. It is generally assumed that the lower temperature leads to reduced rates of oxidative DNA damage and hence to fewer mutations in resulting sperm [1,5]. A second concept relates to the fact that sperm are stored, often over many days or weeks, in the epididymis, particularly the cauda epididymis, which resides at the coolest location within the scrotum [6]. A lower temperature would lead to reduced metabolic rate and oxidative damage in these stored sperm. Linking these concepts is one that suggests that the lower temperature provides a selection process for the best adapted sperm able to confront the metabolic stresses of ejaculation and fertilization [7]. Subsequently, in a number of species, the exteriorized testes in their scrotal sacs have acquired behavioural importance as signals of sexual prowess, leading to the evolution of exaggerated colours and dimensions. In some species, such as dogs, the scrotum can become hairless and acquire a dark coloration to aid heat radiation (Fig. 1), emphasizing again the physiological importance of a cool scrotum. Besides coloration, the usual mechanism of scrotal cooling in high ambient temperatures appears to be by sweat evaporation from the scrotal surface, though the study by Yaeram et al. [3] with mice suggests that even very mild elevation of ambient temperature, in this case exposure to 36°C for 12 hours per day, can impair spermatogenesis.

## Scrotal pathology and temperature

There are two common pathologies associated with testicular heating: cryptorchidism and varicocele. In cryptorchidism, due to a variety of causes, one or both testes may be retained within the abdominal cavity or within the inguinal canal rather than descending normally into the scrotum, which in humans logically occurs at birth. Testicular descent in humans is separated into two major phases [8,9]. The first phase, which occurs during pregnancy, is characterized by a relocation of the testes from a more dorsal perirenal position to one in the inguinal region of the body cavity. This is achieved by dissolution, under the influence of androgens, of the cranial suspensory ligament securing the fetal testes dorsally, and most importantly a growth and development of the ventrally located gubernacular ligament [9]. This is the result of a combination of androgen action, some effect of the peptide Anti-Mullerian Hormone (AMH), and most importantly the relaxin-like male hormone, Insulin-like peptide 3 (INSL3) [9-11]. The role of AMH in testicular descent is unclear, whilst there appears to be a small in vitro effect on gubernacular growth [11], most studies show no influence of this hormone [10,12]. How the testes enter the scrotum is not well understood, though a considerable body of evidence shows that the majority of children whose testes are descended into the inguinal region, but cannot progress further, can be successfully treated by stimulating the hypothalamo-pituitary-gonadal axis, either by GnRH (gonadotropin releasing hormone) analogues or by hCG (human chorionic gonadotropin) [8,13,14]. This is usually interpreted to imply that also this phase is under androgen control, but since this treatment serves to stimulate Leydig cell differentiation and activity, it might also imply that other Leydig cell products, of which INSL3 is one, might also be involved.

**Figure 2:**
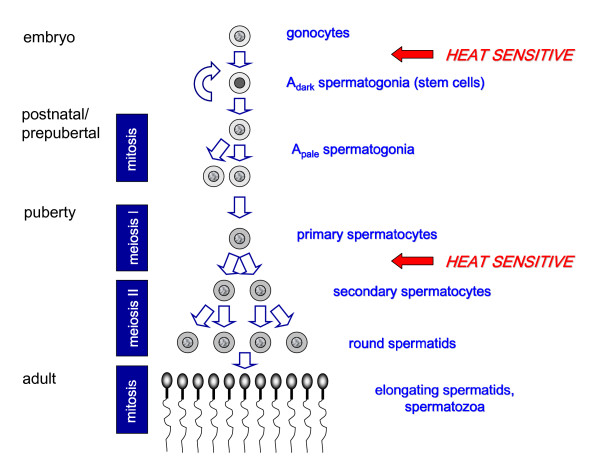
Diagram to illustrate spermatogenesis in the human and those steps which appear to be particularly heat-sensitive. It is largely unclear how a small increase in temperature induces apoptosis. In the adult, both germ cells and Sertoli cells appear to be responsive to heat and show changes in the expression of, for example, heat shock proteins [17-19].

The consequences of cryptorchidism are now well established. A failure to descend the testes in early childhood leads to reduced testicular function, particularly reduced sperm production and infertility [14,15]. More significantly, there is also a correlation with the development of testicular cancer [16]. A large number of studies in animals with induced cryptorchidism, or local testicular heating as adults, show that the process of spermatogenesis is exquisitely sensitive to temperature (Fig. 2), with increased germ cell apoptosis (mostly spermatocytes and spermatids) a consequence of only relatively short periods of mild testicular heating (reviewed in [2]). The neonatal and prepubertal periods were previously considered relatively inert, so that a surgical correction of an abdominal testis (orchidopexy) could be timed for any age prior to the onset of puberty and the commencement of spermatogenesis. Recent observations show that this is incorrect, and that abdominal temperature can markedly influence the number of spermatogonial stem cells that are available to generate all sperm in later life [20]. It appears that there is an inverse relationship between the number of germ cell stem cells (A_dark _spermatogonia) in testis biopsies and the age at which orchidopexy was performed [20], and as a consequence there is a relationship also to sperm production in the later adult. The earlier the testes reach the scrotum after birth, the better the chances of full spermatogenesis as an adult.

The detailed mechanisms operating at a cellular or molecular level are unclear. This is partly due to the fact that it is not possible to distinguish between effects due to unnatural heating of the postnatal testis, and factors which led to cryptorchidism in the first place. So, for example, whilst it appears that A_dark _spermatogonia are negatively affected by postnatal warming, these cells or their progenitor gonocytes may also be targets of the same endocrine disruptors that can affect male differentiation *in utero*, leading also to gonocyte transformation and the formation of carcinoma-in-situ (CIS) cells, which are the seeds of later seminomas [21]. Similarly, a common feature of the perinatal cryptorchid testis are poorly functioning Leydig cells, and hence reduced androgen levels in the so-called perinatal "minipuberty" [22]. It is known that endocrine disruptors such as phthalates can lead to both reduced Leydig cell function and to cryptorchidism [23-25]. Therefore, at this stage in time, we cannot state what are pure effects due only to unnatural testicular heating in early childhood, and what may be effects linked to the original cause of the cryptorchidism.

To complicate the issue further, there may be circumstances where an increase of testicular temperature to that of the abdomen appears to be advantageous. For example, in the *jsd/jsd *mouse, which has impaired spermatogenesis due to a mutation in the *mUtp14b *gene, retention of the testis in the abdomen actually rescues the arrest of spermatogonial differentiation, thus partly restoring spermatogenesis [26]. However, more extensive studies show that the higher temperature is actually causing a subtle androgen-control system to be down-regulated, and that the activated androgen receptor is mediating the arrest of spermatogenesis in this mutant [27]. Thus this mutant does not contradict the generally negative effects of abdominal temperature on testis function.

In varicocele, there is large varicose distension of venous blood vessels within the scrotum, usually associated with increased blood flow [28,29]. One physiological consequence of this, besides increased oxidative stress, is also increased local temperature of the testis, and hence impairment of spermatogenesis [28-31].

## Lifestyle factors and scrotal function

It has long been a source of investigation and speculation that the adoption of certain styles of clothing and posture can influence testicular function, and in particular spermatogenesis. The importance of clothing style and its tightness remains controversial (reviewed in [32,33]), as also is the influence of hot baths, saunas, etc. However, given that the natural cooling methods for the scrotum rely on sweating and vasomotor changes [34], it is now very clear that clothing in combination with behaviour or posture can have significant effects. Normal scrotal temperature (the external surface of the scrotum) is approximately 34°C in a normally clothed man walking about or maintaining a loose stance, and it has been estimated that testicular temperature within the scrotum is between 0.1 and 0.6°C higher than this [32,35,36]. Clothing itself appears to contribute about 0.5–1.0°C [37], compared to being naked. Clothed and sitting down with thighs apart raises scrotal temperature to about 35 C, whereas sitting with thighs together quickly allows scrotal temperature to rise to above 36 C, i.e. to abdominal temperature within the testis [36]. Several studies have now shown that men with predominantly sedentary occupations [36], or who spend considerable time driving a vehicle [38,39], have higher average scrotal temperatures and consequently lower average sperm production or reduced fertility. Another situation where scrotal temperatures become elevated is during sleep, when bedclothes and lack of movement prevent ventilation. Men with oligozoospermia or oligoasthenozoospermia who were allowed to sleep with a small apparatus which permitted nocturnal scrotal cooling showed significant improvements in semen quality over a period of 12 weeks [32,33]. This time is required to allow positive effects on all stages of spermatogenesis, including stem cells to become manifest in the ejaculate. Taken together, these studies all indicate that a more sedentary lifestyle can and most likely does cause a significant impairment in sperm production and quality.

All of the preceding studies relate to testis function in the adult. There is far less known about the impact of lifestyle factors on the important prepubertal and perinatal stages of reproductive development. This is the period when the endocrine axes become established and the functionality of individual cell components of the testis is determined. Besides the generally negative effects of sedentary habits on children in the context of their later developing symptoms of obesity and metabolic syndrome (e.g. [40]), continual over-heating of the testes are likely to impact also on the establishment of spermatogenesis during adolescence. Possibly more significant than this, however, are the consequences on reproductive parameters of the wearing of nappies (diapers) in infancy. Nappy use leads to a long-term increase in scrotal temperature of 1–2 C, compared to air-exposure [41,42]. There appears to be little difference between the use of modern disposable nappies and old-fashioned cloth nappies used together with a plastic cover. If these become wet or soiled, then a further increase in scrotal temperature may be recorded [42]. Dry cloth nappies without any covering only appear to have a small effect on scrotal temperature [42]. Babies are often sedentary or asleep for long periods and nappies may become soiled or wet for much of this time. Nappy usage can extend for several years at a time when the testes may be establishing their reproductive potential. If we consider the risks to the A_dark _spermatogonial stem cell population that may be incurred by a delay in surgically descending cryptorchid testes (see above), then the usage of modern disposable nappies may also be incurring similar long term consequences to the reproductive potential of future men. Since cryptorchidism is associated with testicular cancer, one recent study has looked at whether nappy-wearing has consequences in terms of testicular cancer risk [43]. In this retrospective study, no association was found. However, as mentioned above, there is no evidence to show that it is testicular heating rather than some common intrauterine cause which leads to CIS cells and resultant testicular cancer.

## The role of the epididymis

**Figure 3:**
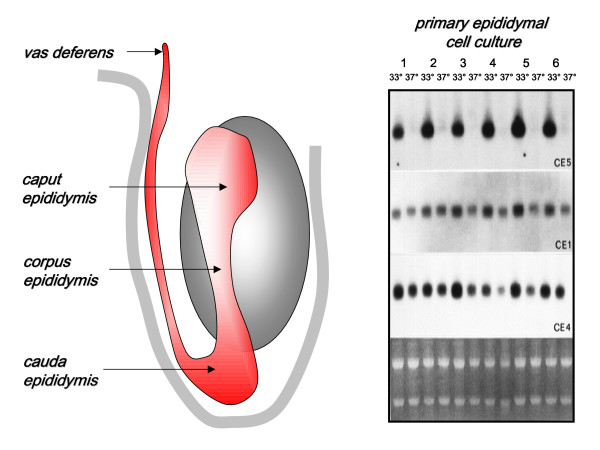
Effect of heating on epididymal cell function. The left panel indicates the gross anatomy of the epididymis within the scrotum. Sperm exit the testis via the excurrent ducts, first entering the initial segment of the epididymis, before traversing successively through the caput, corpus and cauda epididymis, where successively different sets of epidiymal gene products interact with the transiting sperm. The right panel shows northern hybridizations of mRNA extracted from six different independent epididymal epithelial cell cultures subjected to either scrotal (33 C) or abdominal (37 C) culture temperatures. Panels indicate from top to bottom gene transcripts for the epididymis-specific CE5 (CD52; the principal sperm-surface antigen), CE1 (also known as the lipocalin Niemann-Pick C2), and CE4 (a putative extracellular protease inhibitor) genes. The bottom panel is a loading control indicating by ethidium bromide staining the intensities of the 18S and 28S ribosomal RNA bands. Reproduced with permission from Pera et al. [45] (*Copyright 1996, The Endocrine Society*).

In most mammals, sperm leaving the testis are unable to fertilize an oocyte, but first need to undergo a maturation process, which occurs in the epididymis. This is also likely to be the case in humans. The epididymis comprises a highly specialized tubular structure subdivided essentially into three major regions: caput, corpus and cauda. Sperm leave the testis via the excurrent ducts and enter first the initial segment of the caput, followed by the caput proper, and subsequently the corpus and cauda epididymis. The nature of the epididymal maturation process is unclear, though certainly involves the modification of the sperm membrane by interaction with epididymal products, and the acquisition of the ability to become capacitated (reviewed in [44]). Differential cloning and expression studies show that the epithelium of the epididymis produces large amounts of highly specific gene products, which are regionally specialized, and many of which interact with and modify the surface of sperm in transit. Anatomically, the cauda epididymis is located in the most ventral and coolest region of the scrotum. This is the region where sperm are stored for periods of days to weeks, and has given rise to the notion that one of the reasons for the evolution of the scrotum was to provide optimal conditions for sperm storage [6]. Using epididymal epithelial cell cultures, it was shown that one of the major gene products of the epididymis, the major sperm surface antigen CD52, was almost completely and specifically inhibited by switching culture temperature from scrotal temperature (33°C) to abdominal temperature (37°C) (Fig. 3) [45]. This experiment showed that the temperature sensing mechanisms regulating epididymal gene expression are exquisitely sensitive, and that probably artificial elevation of scrotal temperature is likely to have negative effects not only on spermatogenesis in the testis but also on sperm maturation and storage processes within the epididymis.

All studies to date relate to experiments carried out in adult-type systems. There is no information about effects of scrotal temperature on the early development of the epididymis or its functions, or whether long-term exposure to higher temperatures early in life could elicit adaptive processes of benefit in adulthood.

## Conclusions and consequences

There is currently worldwide concern over possible effects of endocrine disrupting agents, with much evidence pointing to an impact by such xenobiotics on the developing fetus *in utero*. In the context of male reproduction there appears to be a recognized association between xenobiotic exposure and traits such as hypospadias, cryptorchidism, CIS and testicular cancer, and reduced anogenital distance [25,46]. In animal experiments, where rats have been given high doses of phthalates, this extends also to reduced adult sperm production. Whether xenobiotic exposure of the male fetus is responsible for the sometimes considerably reduced human sperm counts in some European countries is still debatable. A key feature of traits such as low sperm counts is that the trends are consistently increasing over long periods of time, indicating clear cohort effects, and regionally different rates of change. Together, these features point to an environmental influence, which is associated with increased industrialization. More recently, a similar trend has been reported in the context of the Massachussetts male ageing study, for the total circulating testosterone. This appears to be declining in ageing men in a cohort-dependent manner, which is independent of other lifestyle confounders [47].

The accumulated evidence is highly compelling that the scrotum has evolved in order to provide a local environment for sperm production, maturation and storage that is several degrees cooler than core body temperature. A failure to cool the scrotum adequately appears to be associated in the adult with impaired spermatogenesis, in young boys possibly also to a reduced gonadal stem cell population. Lifestyle factors such as clothing, posture, sedentary behaviour, and the wearing of nappies by babies all seem to predicate a worsening of male reproductive parameters, possibly contributing amongst other factors to the secular trends observed in sperm counts across the globe.

However, most of the evidence is circumstantial, and at least one author [48] has suggested that a direct proof of such effects remains "untestable" in the context of evidence-based medicine. The reasons for this are largely due to the uniqueness of the human situation. There are few experimental animal models which can be considered comparable or appropriate for the human. We undergo a period of relative testicular quiescence of more than 8 years from birth until the beginnings of puberty, when possible negative influences on scrotal temperature could impact subtly upon stem cell populations, as well as during the whole adult lifespan. Rodent models exhibit a comparable prepubertal period of barely 2 weeks, whilst other models such as sheep or dogs can extend this to a period of up to 2–3 months. These are time periods which are biologically not comparable. Add to this that the human species appears to have a fundamental, genetic impairment of spermatogenesis compared to domestic and laboratory species which have been bred for fertility and fecundity. There are indeed a number of mutations in fertility genes in the human, which are considered important in other species [49,50], suggesting that humans as a species are essentially subfertile, and thus more predisposed to subtle negative environmental influences. Epidemiological studies also appear to be highly problematic, largely because of an absence of appropriate matched control groups, i.e. who do not wear clothes, or wear no nappies of any kind, etc.

We are therefore confronted with a difficult problem. Until direct rather than circumstantial evidence can be produced, current lifestyle habits and conveniences will prevail, probably to the detriment of our reproductive capacity. This is the core tenet of evidence-based medicine. The challenge therefore must be met to devise the experiments which will convincingly provide that evidence or refute the possibility.

## Competing interests

The author(s) declare that they have no competing interests.
